# First person – Skylar Graves

**DOI:** 10.1242/bio.062521

**Published:** 2026-03-04

## Abstract

First Person is a series of interviews with the first authors of a selection of papers published in Biology Open, helping researchers promote themselves alongside their papers. Skylar Graves is first author on ‘ Changes in temperature and precipitation drive shifts in mean flowering timing of tropical plants from, 1960–2021 across seven locations’, published in BiO. Skylar is currently a PhD student (graduating May 2026) in the lab of Erin Manzitto-Tripp at the University of Colorado Boulder, USA, measuring changes in tropical flowering, which can be used as a measure of the impact of climate change on an ecosystem.


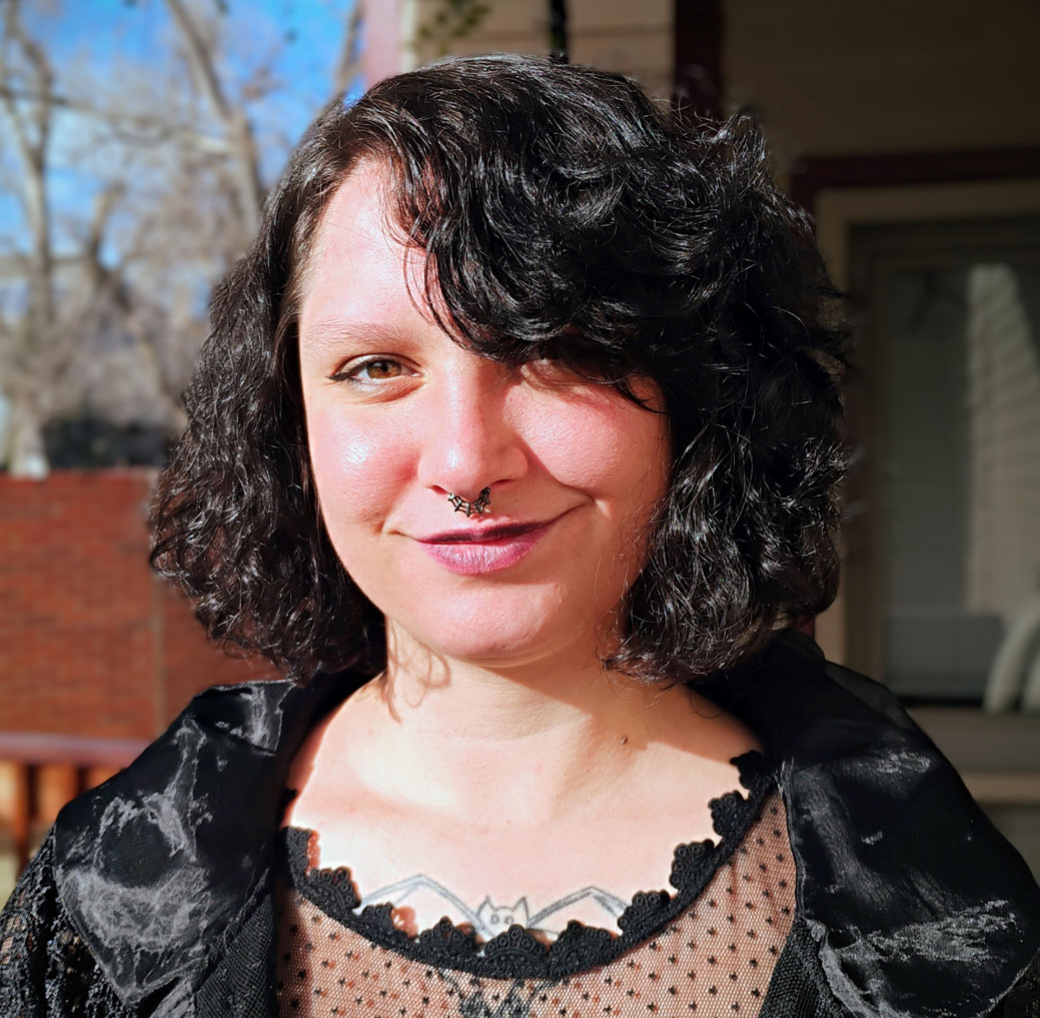


**Skylar Graves**

**Describe your scientific journey and your current research focus**

I fell in love with botany after traveling to Montserrat to study the pioneer species that first colonised the landscape after the volcanic eruption. That passion led me to Dr Tripp's lab for my graduate studies; however, as I applied 2019, my start date was August 2020. This made field work difficult, and I shifted to highlighting my passion in herbaria, and the extensive uses of herbarium specimens. I discovered phenology and sought to explore the changes in flowering phenology in the understudied tropics, using and showcasing herbarium specimens and GBIF. I have found – by studying the impacts of climate change – I am able to have a real-world impact with my research. I hope to expand my work into partnering with conservation organisations, to provide increased data to support evidence-based conservation practices, from the flora up the food chain.

**Who or what inspired you to become a scientist?**

I have always been drawn to biology, and my family supported this passion by encouraging me to explore human biology, with a goal of going into medicine. However, when I reached my undergraduate studies and took my first botany class, I was drawn to how much I didn't know. The euphoria of learning so much so fast and seeing the world around me with new (plant-blindness free) eyes was intoxicating, and I found myself exclusively wanting to study plants. This experience was quickly followed by the opportunity to travel to the tropical island of Montserrat to study the pioneer species that colonised the volcanic ash, only a few years after the last eruption. The incredible biodiversity in tropical latitudes captivated me, and the rest is history.

**Figure:**
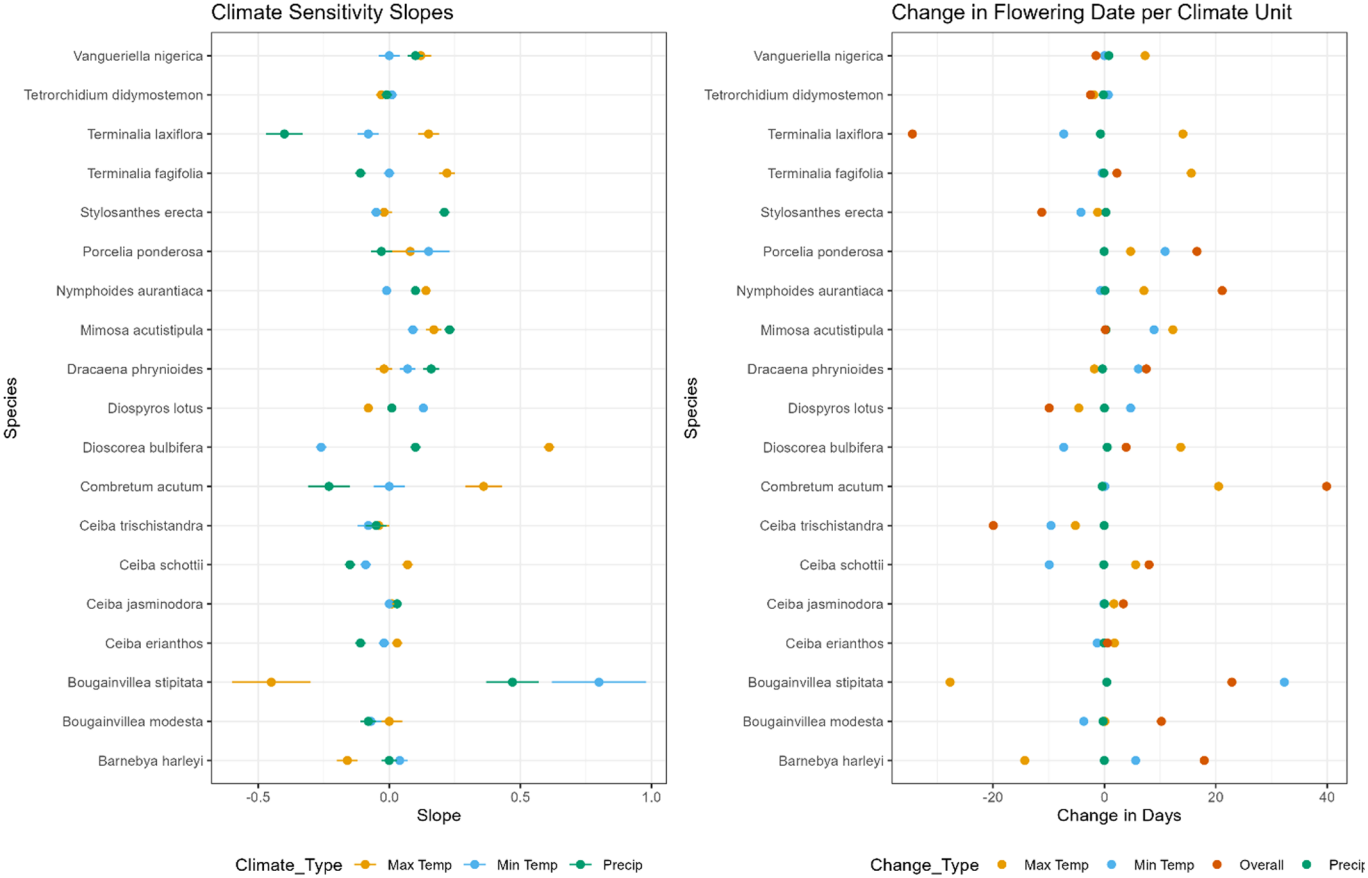
**(Left) Slope of regression representing change in flowering date in relation to change in average monthly maximum temperature (yellow), average monthly minimum temperature (blue) and total monthly precipitation (green) for each species, with 95% confidence interval error bars**. (Right) Change in flowering date per change in °C of maximum temperature (yellow), minimum temperature (blue) and mm precipitation (green) and overall change in the combined effects of all three aforementioned variables (red).

**How would you explain the main finding of your paper?**

We found the impacts of changing temperature and precipitation on flowering timing in tropical latitudes is comparable to the changes found in temperate latitudes. The consistent warm temperatures year-round do not insulate these species from the impacts of climate change.

I hope among the impacts of my research is an increase in the perceived value of herbarium specimens

**What are the potential implications of this finding for your field of research?**

I hope among the impacts of my research is an increase in the perceived value of herbarium specimens. This work highlights herbarium specimens as more than taxonomic tools. Herbarium specimens make up a massive source of data, far greater in both geographic and temporal scale than any one researcher can hope to achieve in their lifetime. I hope studies like mine can be persuasive for increased funding of herbaria and their digitisation worldwide.

**What's next for you?**

I am finishing my PhD in May 2026 and looking for post-doc opportunities in Europe. I am looking forward to exploring connections to conservation organisations and taking the impact of my work outside of simply publishing it but by seeing real-world impact. With the rapid advancement of climate change, I feel it is crucial to do everything I can to have even a small positive impact.
